# A New Termitophilous Genus of Paederinae Rove Beetles (Coleoptera, Staphylinidae) from the Neotropics and Its Phylogenetic Position

**DOI:** 10.1007/s13744-022-00946-x

**Published:** 2022-02-17

**Authors:** Dagmara Żyła, Amalia Bogri, Aslak Kappel Hansen, Josh Jenkins Shaw, Janina Kypke, Alexey Solodovnikov

**Affiliations:** 1 Museum and Institute of Zoology, Polish Academy of Sciences, Warsaw, Poland; 2 Leibniz Institute for the Analysis of Biodiversity Change, Zoological Museum, Hamburg, Germany; 3 Natural History Museum of Denmark, Univ of Copenhagen, Copenhagen, Denmark; 4 Key Laboratory of Zoological Systematics and Evolution, Institute of Zoology, Chinese Academy of Sciences, Beijing, China

**Keywords:** Taxonomy, Systematics, Phylogenetics, Rove beetles, Inquiline, South America

## Abstract

We describe a new genus and species of Paederinae rove beetles, *Ruptor cordatus* gen. et sp. nov., which lives in the arboreal nests of the termite *Labiotermes labralis* (Holmgren, 1906) in the Amazon lowlands of Peru. The morphology of *Ruptor* gen. nov. is highly derived, apparently due to its close association with the termite host, and thus, morphologically, the genus cannot be classified further than Lathrobiini *incertae sedis*. In order to address the sister-group relationships of *Ruptor* gen. nov., we conducted a molecular phylogenetic analysis based on seven gene fragments. The analysis indeed resolved the genus as a member of the tribe Lathrobiini and placed it nested within the informal clade of ‘Medonina and allied taxa’. We provide a morphological comparison of the new genus with all known myrmeco- and termitophilous representatives of the subfamily, and to the extent possible, we illustrate other relevant and poorly known Neotropical Paederinae inquilines.

**Supplementary Information:**

The online version contains supplementary material available at 10.1007/s13744-022-00946-x.

## Introduction

Paederinae is one of the most diverse and abundant subfamilies of the family Staphylinidae (rove beetles), comprising more than 220 genera and 7600 species (Żyła et al. 2021). It is a globally distributed subfamily, well represented in higher latitudes but much more diverse in the tropics. Notably, 99 genera and 1684 described species are confined to the Neotropical region alone (Asenjo et al. 2019). The true Paederinae diversity is expected to be even higher in this region since the main body of the taxonomic work hitherto conducted on that group was limited to the Holarctic and, to a lesser degree, the Neotropical, Indomalayan and Afrotropical fauna.

Outdated subtribes and many poorly delimited genera in Paederinae, i.e. taxonomic groupings not coinciding with phylogenetic lineages, greatly hinder the taxonomic work on this subfamily in the Neotropics and other poorly studied regions. The situation gets even more complicated when one considers highly specialised myrmecophiles or termitophiles, in which their peculiar morphological adaptations to social parasitism lead to odd habitus and drastic modifications that mask diagnostically or phylogenetically valuable morphological characters.

**Fig. 1:**
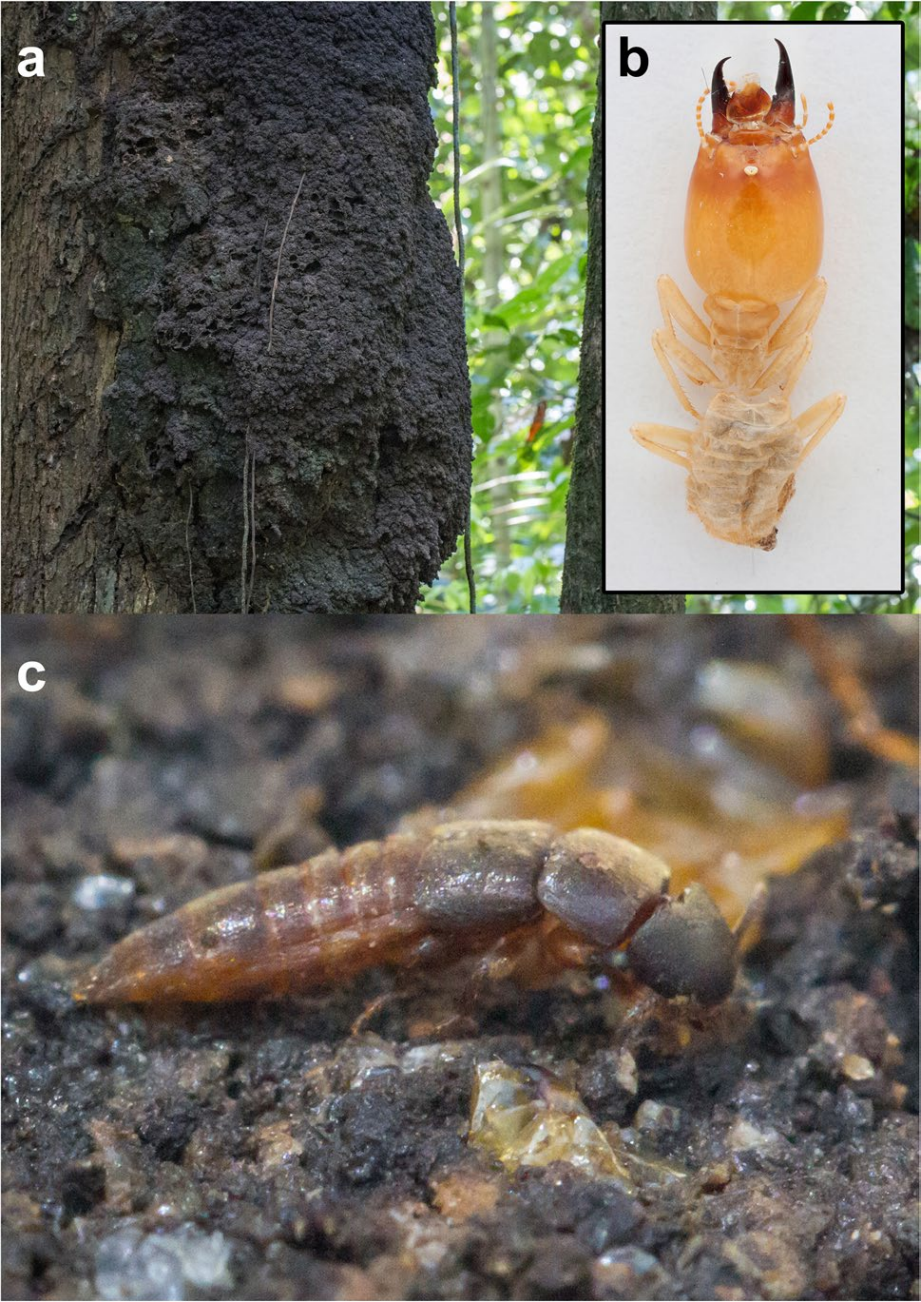
Rove beetle *Ruptor cordatus* gen. et sp. nov. and its termite host *Labiotermes labralis* (Holmgren, 1906). **a** Arboreal termite nest of *Labiotermes labralis*; **b** worker specimen of *Labiotermes labralis*; **c** live specimen of *Ruptor cordatus* photographed on the disassembled termite nest debris before being collected

**Fig. 2:**
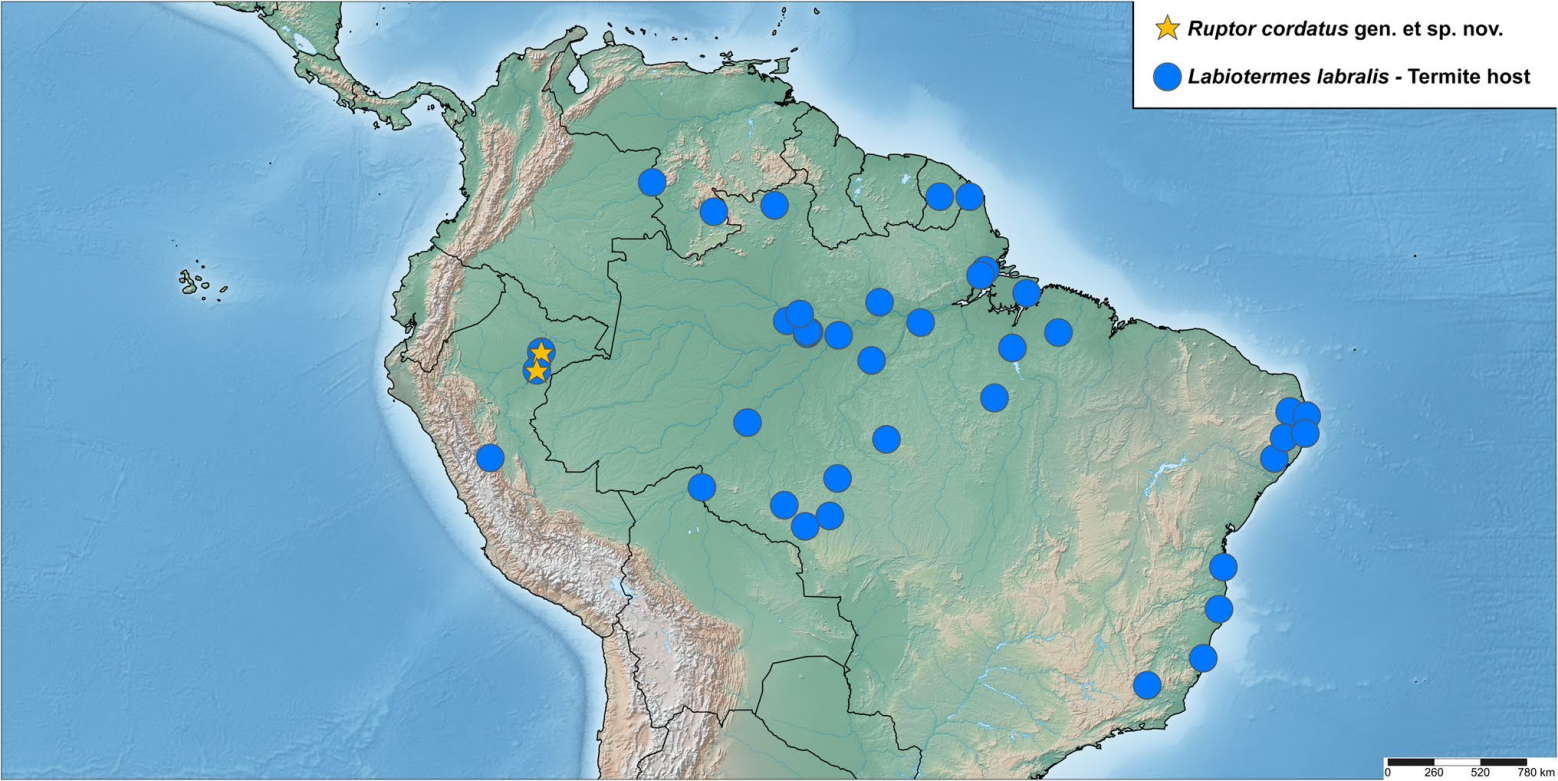
Distribution of *Ruptor cordatus* gen. et sp. nov. (orange star) and its termite host *Labiotermes labralis* (Holmgren, 1906) (blue circle), the latter based on Constantino et al. (2006)

Within Staphylinidae, Paederinae is one of a few subfamilies where social parasitism, in particular myrmeco- and termitophily, has evolved. Inquiline paederines are especially diverse in the Neotropics (Seevers 1957; Kistner 1979). Thus, we faced a complex task in which we had to identify four specimens of an odd-looking termitophilous paederine species, not matching any known genus, which we (A.K.H., J.K. and A.S.) collected in the nests of the termite *Labiotermes labralis* (Holmgren, 1906) (Fig. 1) in the Amazon lowlands of Peru (Fig. 2).

The objective of this paper is to describe a new genus and species of Paederinae rove beetles. Given the emerging backbone phylogeny of Paederinae based on total-evidence data (Żyła et al. 2019, 2021), as well as the availability of the DNA-grade material for this newly collected species, we decided to conduct a phylogenetic analysis to determine the lineage where this new taxon belongs and complement its taxonomic treatment.

## Material and methods

### Deposition of taxa

All specimens of the new species were acquired by the authors during a field trip to the Peruvian Amazon under the permit number RDG 0328–2017-SERFOR-DGGSPFFS/RDG 356–2017-SERFOR-DGGSPFFS. Following the conditions of the permit, the male holotype and a female paratype are deposited at the Museo de Historia Natural de la Universidad Nacional Mayor de San Marcos in Peru (MHN-UNMSM). Two remaining female paratypes are kept at the Natural History Museum of Denmark in Copenhagen (NHMD). Information on the deposition of taxa used for phylogenetic analysis is given in Supplementary Table 1.

### Microscopy and illustrations

The specimens were examined using Leica M205 C and Leica M125 stereoscopes. All photos were taken with either a Canon EOS 5D Mark III digital camera with a Canon MP-E 65 mm Ff2.8 1–5 × macro lens or Canon EOS 6D fitted on a Zeiss Axioskop slide microscope at 10 × both using the remote shooting function of EOS Utility v3.4.30.0. Photostacking was achieved using Zerene Stacker (Zerene Systems LLC, 2012) and the photos were edited in Adobe Photoshop 2021. Line drawings were made using images or camera lucida sketches that were digitally inked in Adobe Illustrator 2021.

### Taxon sampling and outgroup for phylogenetic analysis

In total, 51 taxa were included in the dataset (Supplementary Table 1). We sampled all currently recognised tribes and subtribes of Paederinae with a focus on Lathrobiini where the newly described genus was suspected to belong based on morphological observations. We added representatives of closely related subfamilies, namely Staphylininae and Xantholininae (sensu Żyła and Solodovnikov 2020) as the closer related outgroup and representatives of Oxyporinae, Tachyporinae and Mycetoporinae as the more distantly related outgroup. Unfortunately, we were unable to include many taxa which would ideally be represented in such an analysis based on the morphological examination of our target termitophilous species. This especially applies to the many species and genera currently placed in Medonina, because they are unavailable as DNA-grade material.

### GenBank data

To construct our molecular matrix, we used seven gene fragments: the nuclear protein-encoding genes carbamoylphosphate synthetase (CADA and CADC), topoisomerase I (TP), arginine kinase (ArgK), wingless (Wg), the mitochondrial protein-encoding cytochrome c oxidase I (COI) and the nuclear ribosomal 28S. The GenBank accession numbers of all sequences are given in Supplementary Table 1. All sequences, except for the new genus, were already used in Żyła et al. (2021).

### DNA extraction, amplification and sequencing

For the DNA extraction of *Ruptor* gen. nov., we used the Qiagen DNeasy Blood and Tissue kit (Venlo, Netherlands) using the protocol for animal tissue with a prolonged lysis time (12–16 h). The abdominal apex was removed from the specimen and used for the non-destructive extraction (including segment VIII, the genital segment and the aedeagus). The extract was stored in a – 20 °C freezer for the period of the project, while the physical voucher (holotype) was pinned on a card mount with glycerin filled microvial for the genital segments and aedeagus which is deposited at MHN-UNMSM. We used the same protocols as in Żyła et al. (2021) for amplification, sequencing, sequence editing and assembly. Polymerase chain reactions were performed in 25 μL reactions. The reaction consisted of 2 μL of DNA extract, 4 μL of 5 × HOT FIREPol Blend Master Mix Ready to Load With 10 mM MgCl2 (Solis BioDyne), 0.5 μM of each primer and 15 μL distilled water. In the case of a second reaction for the genes using nested PCRs, only 1 μL of DNA extract was used, with the analogous increase in distilled water. Sanger sequencing and purification of target genes was done by Macrogen (Amsterdam, Netherlands).

### Sequence alignment

Sequences were newly aligned in Geneious v9.1.7 (Biomatters Ltd, Auckland, New Zealand) using the MAFFT plugin v1.3.6, based on MAFFT (Katoh et al. 2002). 28S was aligned using the E-INS-i algorithm of MAFFT and ambiguously aligned regions were identified and removed with the server version of Gblocks (Talavera and Castresana 2007). We allowed gap positions within the final blocks and less strict flanking positions but did not allow many contiguous nonconserved positions. The resulting 28S alignment was 795 bp and had very few, scattered and usually single-nucleotide gaps. Individual gene alignments were concatenated with the ‘concatenate’ function of Geneious. The concatenated sequence alignment is provided in Supplementary File 1 in fasta format.

### Phylogenetic analysis

A matrix of molecular data (4566 bp) for the total number of taxa under study (51) was analysed using the Bayesian Inference method. The alignment was initially partitioned by gene and, for protein-encoding genes, by position. The optimal partitioning scheme and the corresponding models of nucleotide evolution were determined by PartitionFinder v. 2.1.1 (Lanfear et al. 2016) using the Bayesian Information Criterion. All models were considered, branch lengths were unlinked and the search was set to the ‘greedy’ algorithm (Lanfear et al. 2012). Bayesian inference (BI) was conducted in MrBayes v3.2.6 (Ronquist et al. 2012) running on CIPRES Science Gateway v3.3. (phylo.org). All analyses used four chains (one cold and three heated) and two runs. The analysis was run for 10 million generations. The script used for the analysis is given in Supplementary File 2 in.nex format. Convergence of the runs was visualised in Tracer v1.7 (Rambaut et al. 2018), and by examining potential scale reduction factor (PSRF) values and the average standard deviation of split frequencies in the MrBayes output. Nodes with (BI) posterior probability (PP) > 0.95 were considered well supported, nodes with PP = 0.90–0.94 moderately supported, those with PP = 0.80–0.89 weakly supported and nodes with PP < 0.79 were considered to be unsupported.

### Identification of the termite host

For identification of the termite, DNA was extracted from the leg of a soldier specimen and from the body of a worker specimen using the above-described extraction protocol. The two specimens were both with the following data ‘PER17-20a, PERU: Amazonia, Loreto region, Maynas Province, Allpahuayo-Mishana NP, 20 km S of Iquitos, 4.IX.2017, 100–200 m, 3°58.725′S 73°25.497′W, rainforest, in termite nest, leg. A. Hansen, J. Kypke, A. Solodovnikov’. The Pat and Jerry region of the COI gene was amplified and sequenced from both specimens using the same protocol as described above. The consensus of two sequences (100% identity, GenBank: OK310885 and OK310886) was compared to the GenBank repository resulting in the best match of 98.0% to a full mitochondrion of *Labiotermes labralis* (GenBank: KY436201). To confirm this identification, these and various other specimens of the termites (both worker and soldiers) were taken through the key in Constantino et al. (2006) resulting in the same determination, *Labiotermes labralis*. Given that this is the only species in the genus that builds arboreal nests and that several methods (genetic and morphological) produced similar results, we feel confident in our identification.

## Results

Subfamily Paederinae Fleming, 1821.

Tribe Lathrobiini Laporte, 1835.

Subtribe *incertae sedis.*

Genus *Ruptor* gen. nov.

Type species: *Ruptor cordatus* sp. nov.

Diagnosis.

**Fig. 3:**
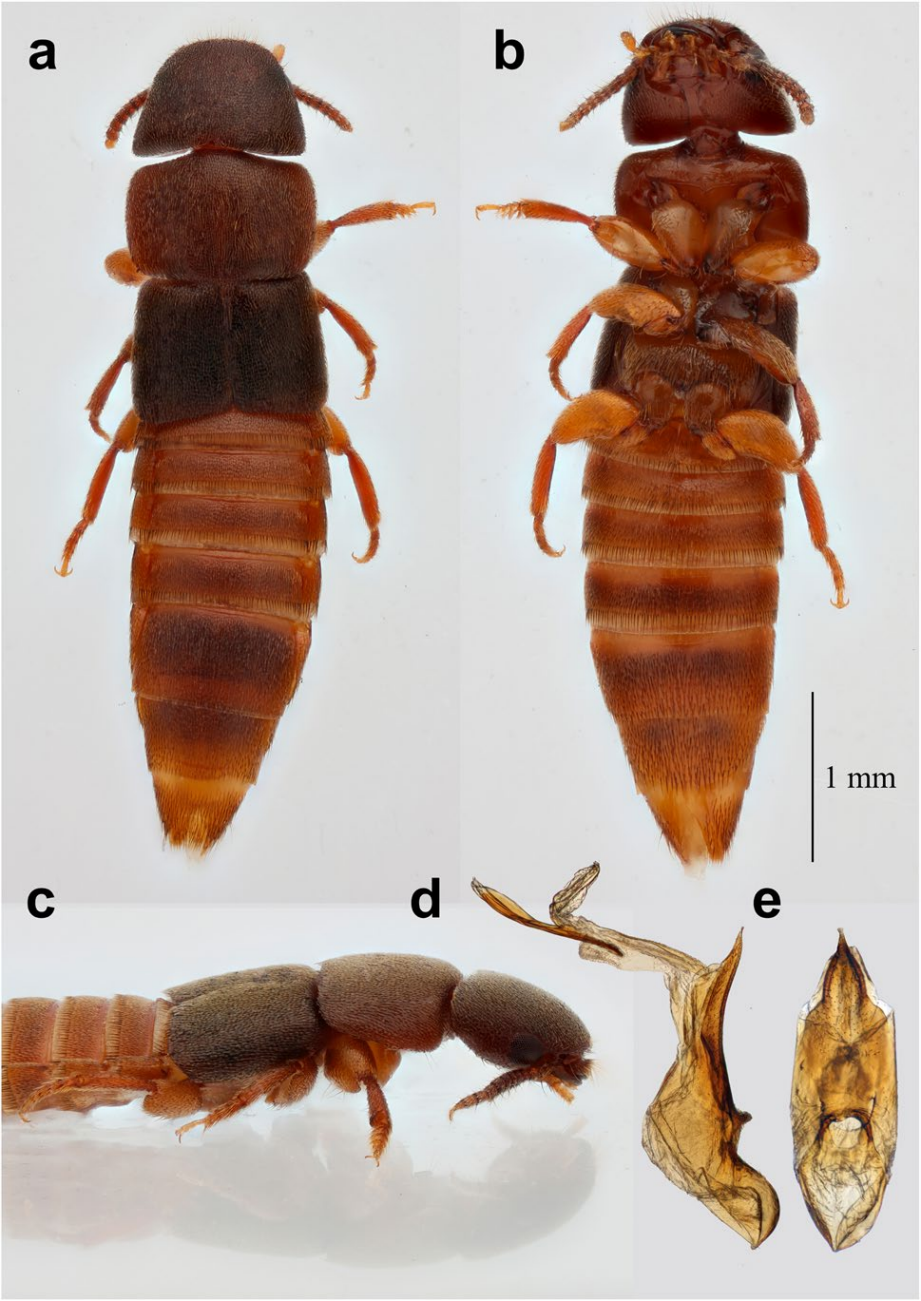
Photos of *Ruptor cordatus* gen. et sp. nov. **a** Habitus in dorsal view; **b** habitus in ventral view; **c** habitus in lateral view; **d** aedeagus in lateral view; **e** aedeagus in parameral view

**Fig. 4:**
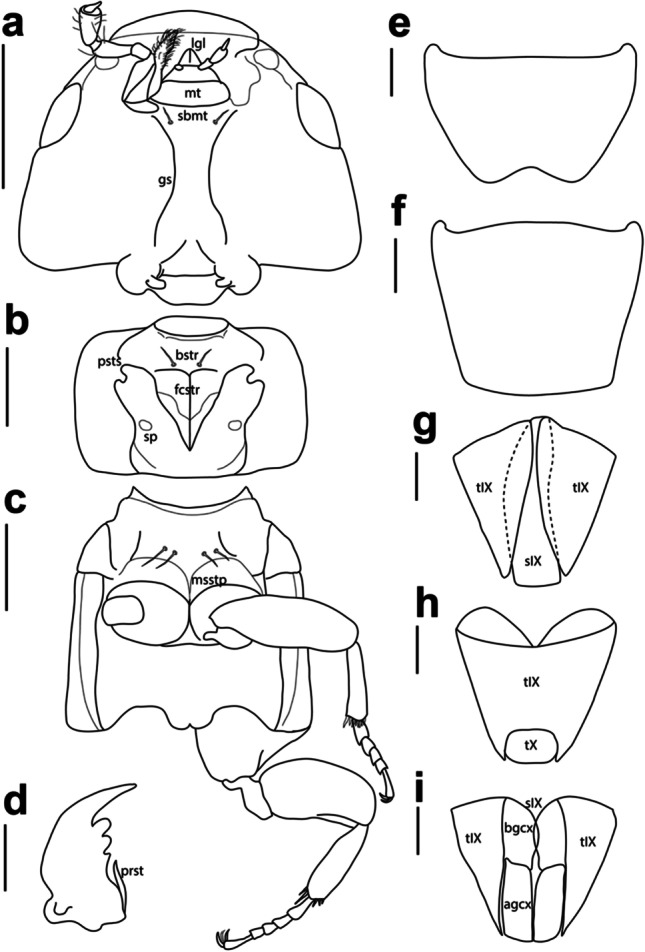
Details of morphology of *Ruptor cordatus* gen. et sp. nov. **a** Head in ventral view; **b** prothorax in ventral view; **c** meso- and metathorax in ventral view; **d** right mandible in ventral view; **e** male sternite VIII; **f** male tergite VIII; **g** male sternite IX surrounded by visible parts of tergite IX; **h** male tergites IX and X; **i** female sternite IX (divided into basal and apical gonocoxites) and visible parts of tergite IX. **a**, **b** and **c** Scale = 5 mm; **d**, **e**, **f**, **g** and **i** scale = 2 mm. Abbreviations: agcx, apical gonocoxite; bgcx, basal gonocoxite; bstr, basisternum; fcstr, furcasternum; gs, gular suture; lgl, ligula; msstp, mesosternal process; mt, mentum; prst, prostheca; psts, prosternal suture; sbmt, submentum; sp, spiracle; sIX, sternite IX; sX, sternite X; tIX, tergite IX; tX, tergite X

*Ruptor* gen. nov. (Figs. 3 and 4a) can be recognised among all Paederinae by the following combination of characters: compact, dorso-ventrally flattened body with dense even cover of setiferous punctures with short pale setae; trapezoid-shaped head with broad frontal area, pronounced posterior angles and small notch on straight posterior margin of head above moderately narrow (ca. 1/3 of head width) neck; in maxillary palps penultimate (third) palpomere slightly longer and wider than second, apical (fourth) palpomere small and acicular; strongly transverse pronotum and notably shortened antennae and legs; protarsi wide, protarsomere 4 simple, not bilobed; protibial combs placed longitudinally (Supplementary Fig. 1a); ctenidium present only on posterior side of hind tibia (Supplementary Fig. 1b); aedeagus without parameres.

Description.

Medium size beetle (total body length ca. 5 mm); body robust and compact, somewhat flattened; body surface with dense but fine irregular punctation. Appendages shortened and robust.

Head trapezoidal, wider than long, widest in posterior part, with distinct posterior angles, tapering towards smooth anterior angles. Antennal insertions and labrum concealed under bulging frons, not visible from above (Fig. 3a, b). Antennae inserted near anterior margin of eye (Fig. 4a), short, slightly pectinate, reaching anterior margin of pronotum. All antennomeres slightly widened apically, without tomentose pubescence, with smaller pale setae all over surface and few longer and bigger setae around apex of antennomere; stem between antennomeres not visible (Fig. 4a–c). Strongly transverse labrum almost covering closed mandibles from above, its anterior margin straight, not notched or dentate (Fig. 4a), highly sclerotised, with multiple, evenly distributed setae. Mandibles short and stout, without ridges, with prostheca extending from base of mandible to first tooth. Left mandible with two teeth; right mandible with three teeth, first one distinctly smaller than next two (Fig. 4d). Maxillae (Fig. 4a): palpomere 1 short, approximately half of length of maxillary palpomere 2, bearing single seta; maxillary palpomere 2 short, expanded towards apex, with few strong and long setae; maxillary palpomere 3 expanded towards apex with denser setation; palpomere 4 (apical) glabrous and acicular, around 1/4 of palpomere 3 length, thin, 1/3 of width of palpomere 3. Labium (Fig. 4a): palpomere 1 widest apically, slightly thinner than palpomere 2; palpomere 2 elongate, widest at middle, slightly longer than palpomere 1, bearing three setae; palpomere 3 (apical) acicular, with few setae, slightly more than half of length of palpomere 2; mentum transverse, slightly concave along anterior margin; submentum with pair of setae; ligula bilobed. Gular sutures widely separated, gula wider in apical and basal portion (Fig. 4a). Eyes of moderate size, without setae between ommatidia, temples 1.5 × longer than eyes. Neck distinct; slightly more than 1/3 of head width.

Prothorax (Fig. 4b) distinctly transverse, widest just anteriad of middle; superior marginal line of pronotum deflexed under its anterior angles, reaching prosternum, not meeting with inferior marginal line; pronotal hypomera broad forming weak postcoxal process; prosternal suture well developed; basisternum with one pair of large macrosetae; furcasternum long (exceeding tip of postcoxal process), narrowly pointed posteriad, with sharp longitudinal carina in its posterior part; thoracic spiracles without distinct perithremes. Elytra without epipleural ridge. Mesoscutellum glabrous with apical third punctate and setose; obtusely pointed apically, with one transversal scutellar ridge before middle of scutellum length. Hind wings fully developed. Mesosternum (mesoventrite) (Fig. 4c) without longitudinal carina; mesocoxal cavities contiguous, mesosternal (mesoventrital) process acutely pointed. Metathorax (Fig. 4c) well developed. All legs relatively short, with broad, enlarged femora; protibia with two large spines at apical margin and two longitudinal combs of setae. Protarsi (Fig. 3c) enlarged in both sexes, protarsomeres 1 to 4 transverse, with adhesive spatulate setae ventrally, protarsomere 3 slightly bilobed. Meso- and metatarsi (Fig. 4c) same in shape, without adhesive setae ventrally; their tarsomeres 1 and 2 equal in length, tarsomere 5 about as long as tarsomeres 2 to 4 combined. One pair of empodial setae on each tarsus, equal or slightly shorter than claws. Hind tibia with ctenidium on posterior side.

Abdomen widest at segment V; segments III to VII with one pair of paratergites on each side; posterior margin of tergites II to VI with fringe of setae; apical margin of tergite VII with palisade fringe. Sternite III with transverse suture acutely pointed medially. Male: sternite VIII with slight medial emargination (Fig. 4e), tergite VIII truncate (Fig. 4f), with usual setation; sternite IX symmetrical (Fig. 4g); lateral tergal sclerites IX fused in one piece without any sutures apically embracing small tergite X (Fig. 4h); aedeagus symmetrical, without parameres (Fig. 3d, e). Female: sternite VIII apically without emargination; lateral tergal sclerites IX and tergite X as in male (Fig. 4h); sternite IX consisting of pair of weakly sclerotised basal and pair of stronger sclerotised apical gonocoxites (Fig. 4i).

For distribution and bionomics see below species description.

Comparison.

**Fig. 5:**
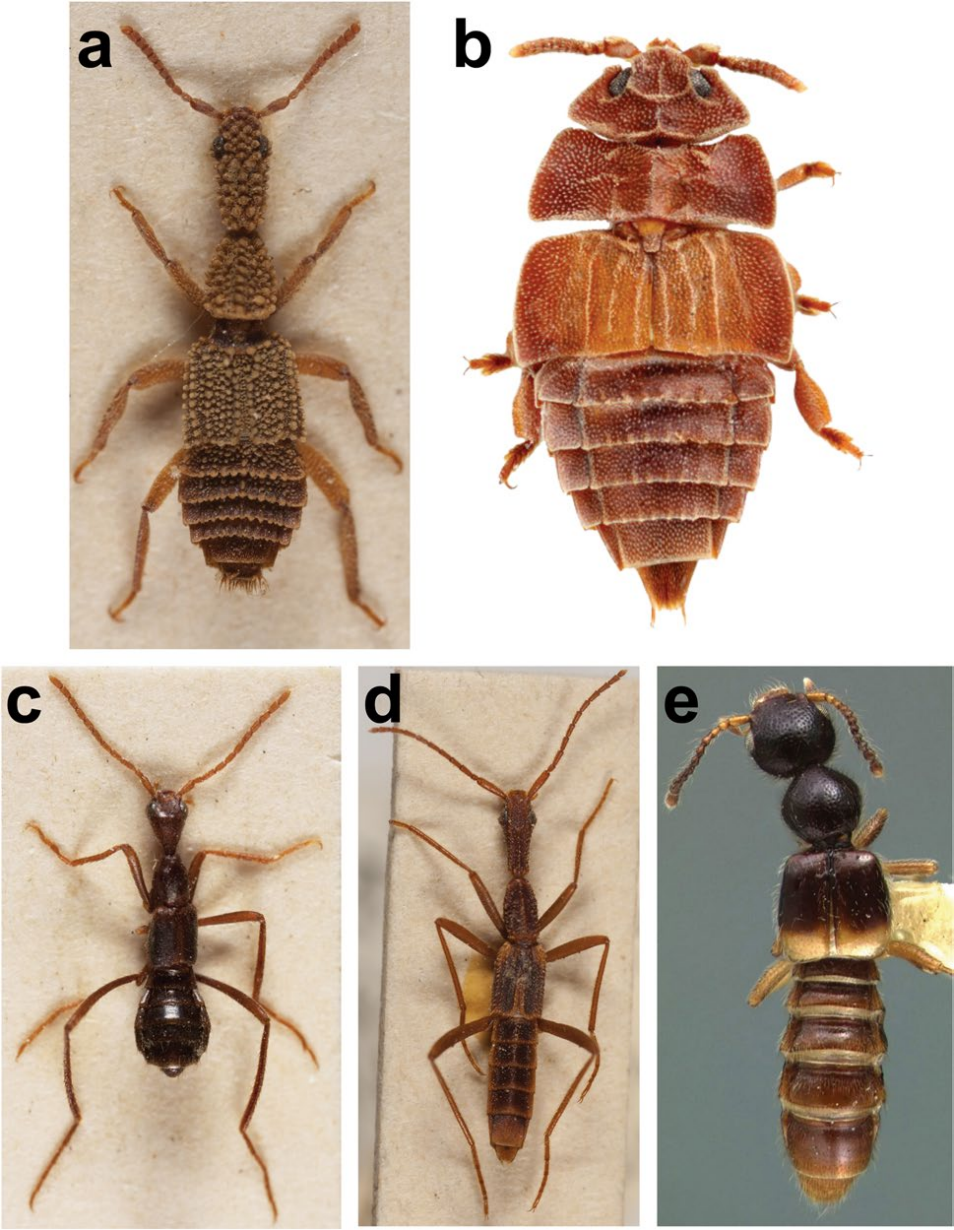
Some little-known Neotropical inquiline Paederinae. **a** *Ecitonides tuberculosus* Wasmann, 1894 (credit Andrea Schomann); **b** *Ecitosaurus* sp. (credit Munetoshi Maruyama); **c** *Mimophites bouvieri* Fauvel, 1904 (credit Andrea Schomann); **d** *Synecitonides phasma* Reichensperger, 1936 (credit Andrea Schomann); **e** *Monista darlingtoni* Blackwelder, 1943 (copyright of Museum of Comparative Zoology, Harvard University (©President and Fellows of Harvard College))

*Ruptor* gen. nov. is rather distinct among all known genera of Paederinae, including specialised termitophilous and myrmecophilous forms (Fig. 5), by its habitus alone. Its compact dorso-ventrally flattened body with short appendages, lack of tuberculose sculpture or carinae on forebody, and head with sharp posterior angles (in dorsal view) combined with transverse pronotum easily tell it apart from other, often poorly known, inquiline (or presumably so) Lathrobiini *incertae sedis* genera, namely *Bolbophites* Fauvel, 1904, *Ecitobium* Wasmann, 1923, *Ecitonides* Wasmann, 1894 (Fig. 5a), *Ecitosaurus* Fischer, 1943, (Fig. 5b) *Ecitotropis* Borgmeier, 1936, *Labidophites* Borgmeier, 1956, *Mimophites* Fauvel, 1904 (Fig. 5c), *Synecitonides* Reichensperger, 1936 (Fig. 5d) and *Monista* Sharp, 1876 (Fig. 5e). The new genus superficially resembles some species of the Neotropical Lathrobiini genera *Attaxenus* Wasmann, 1925, *Paederopsis* Wasmann, 1912 or especially *Dacnochilus* LeConte, 1861, which presumably belong to the recently discovered *Pseudolathra*-Cylindroxystina lineage (Żyła et al. 2021)*.* Within *Dacnochilus*, a widespread Neotropical genus that occasionally occurs in nests of termites and ants, *Ruptor* especially resembles *D. atrus* Jiménez-Sánchez & Galián, 2013 [correct masculine form of the species name should be *ater*], *D. compactus* (Casey, 1905), *D. horridulus* (Casey, 1905), *D. nahuiollinae* Jiménez-Sánchez & Galián, 2013, *D. newtoni* Jiménez-Sánchez & Galián, 2013, or *D. zaragozae* Jiménez-Sánchez & Galián, 2013. However, it readily differs from them by a somewhat rugose (not glabrous) disc of head and pronotum, by the presence of ctenidium only on the posterior side of hind tibia, as well as by the longitudinal placement of the protibial combs. Also, *Ruptor* may distantly resemble members of the subgenus *Eurysunius* Reitter, 1909 of the genus *Astenus* (Astenina). It differs from all Astenina at least by its simple not bilobed protarsomere 4, by its prosternum not being expanded under the front coxae and not fused with the pronotal hypomera, and by its short and stout mandibles. The new genus differs from all myrmeco- and termitophilous Pinophilini and Paederini by having the typical for Lathrobiini small and unmodified apical (fourth) maxillary palpomere; it differs from the myrmecophilous members of Scopaeina by its larger labrum and lack of trichobothrium on the head; it differs from myrmecophilous Stilicina by the wider neck and the prosternum not being expanded under the front coxae and not being fused with the pronotal hypomera.

Etymology: The genus name is a Latin noun of masculine gender meaning burglar or housebreaker. It refers to the social parasitism biology of this taxon which was found in the termite’s ‘house’.

*Ruptor cordatus* sp. nov.

Type material, Holotype: [card mounted, DNA extraction voucher]: ♂, ‘PER17-19a, PERU: Amazonia, Loreto region, Maynas province, Allpahuayo-Mishana NP, 20 km S of Iquitos, 3.IX.2017/100–200 m, 3°57.812′S 73°25.142′W, rainforest, in termite nest, leg. A. Hansen, J. Kypke, A. Solodovnikov/HOLOTYPE *Ruptor cordatus* gen. et sp. n. Żyła et al. des. 2021 [red label]’ (MHN-UNMSM). Paratypes: [card mounted]: 1♀ ‘PER17-19a PERU: Amazonia, Loreto region, Maynas province, Allpahuayo-Mishana NP, 20 km S of Iquitos, 3.IX.2017/100–200 m, 3°57.812′S 73°25.142′W, rainforest, in termite nest, leg. A. Hansen, J. Kypke, A. Solodovnikov/collected with the holotype/PARATYPE *Ruptor cordatus* gen. et sp. n. Żyła et al. des. 2021 [yellow label]’ (NHMD); [disarticulated, in glycerin]: 1♀, ‘PER17-20a, PERU: Amazonia, Loreto region, Maynas Province, Allpahuayo-Mishana NP, 20 km S of Iquitos, 4.IX.2017/100–200 m, 3°58.725′S 73°25.497′W, rainforest, in termite nest, leg. A. Hansen, J. Kypke, A. Solodovnikov/PARATYPE *Ruptor cordatus* gen. et sp. n. Żyła et al. des. 2021 [yellow label]’ (MHN-UNMSM); [in cryovial]: 1♀, ‘PER17-21b, PERU: Amazonia, Loreto region, Requena province, 3 km E of Jenaro Herrera, 6.IX.2017, 100–200 m/4°54.022′S 73°39.104′W, secondary forest/plantation, in termite nest, leg. A. Hansen, J. Kypke, A. Solodovnikov/PARATYPE *Ruptor cordatus* gen. et sp. n. Żyła et al. des. 2021 [yellow label]’ (NHMD).

Measurements of holotype (in millimetres). Length = 4.9; front body length (HL + PL + EL) = 2.27; HW (head width at widest point) = 1.0; HL (head length medially) = 0.6; PW (pronotum width at widest point) = 1.1; PL (pronotum length medially) = 0.78; EW (elytra width at widest point) = 1.18; EL (elytra length from shoulder to hind margin) = 0.89.

## Description

Body colouration varies from pale brown to dark brown; head and elytra always darker; pro- and mesotibia darker than metatibia. Anterior and posterior margins of head, pronotum and elytra evenly covered with short golden setae; rest of head and abdomen mainly with grey setae; disc of head appearing dull due to microsculpture between fine punctation. Antennae: antennomere 1 as long as two following antennomeres; 2 and 3 elongate; 4 to 8 clearly transverse, 9 and 10 slightly transverse, 11 elongate, 1.5 × longer than antennomere 10. Antennomeres 3 to 7 appearing bicoloured, apical third of each antennomere distinctly darkened. Pronotum slightly longer than head. Elytra slightly longer than, and about as wide as pronotum; punctation with punctures larger and more densely positioned than those on pronotum. Aedeagus with narrowly pointed median lobe and characteristically elongate, bifurcate sclerite of internal sac.

## Distribution and bionomics

*Ruptor cordatus* sp. nov. was found inside the chambers of arboreal termite nests (Fig. 1) in the lowland forest of the North-Eastern Peru (Fig. 2). The termite host was confidently identified as a widespread *Labiotermes labralis* (Figs. 1 and 2), the only species in the genus that builds an arboreal nest (Constantino et al. 2006). Based on our sampling, we were able to find *Ruptor cordatus* sp. nov. in roughly every fourth termite nest that was searched. All nests where *Ruptor cordatus* sp. nov. was found were with winged termites, where also a number of other inquiline staphylinids (mostly Aleocharinae) were always encountered.

## Etymology

The scientific name of the new species is a Latin adjective that refers to the cordate shape of the head of the new species due to its characteristic notch in the middle of the hind margin of the head.

### Phylogenetic analyses

PartitionFinder found the following four partitions: (1) 28S + COI2 + ArgK2 + Wg2 + TP2 + CADC2 + CADA2 + COI1 + ArgK1 + CADC1 + CADA1 + Wg1 + TP1; (2) Wg3 + TP3 + ArgK3; (3) CADA3 + CADC3; (4) COI3. For partition 1, SYM + I + G was found to be the best-supported model, for partitions 2 and 3—GTR + I + G, and for partition 4—HKY + G. We moved 28S to a separate partition as this is the only non-protein-coding gene in our dataset and analysed it under the SYM + I + G model. The third codon positions of COI were excluded as it has been suggested that they suffer saturation for deep divergences, which can potentially bias phylogenetic analyses (e.g. Swofford et al. 1996; Lin and Danforth 2004). Since COI3 was excluded, no partition was analysed under the HKY + G model.

The BI analysis reached convergence, with a standard deviation of split frequencies well below 0.01 after 10 million generations. Mixing of the Markov chain Monte Carlo chains was good, effective sample size (ESS) values were greater than 200 for all parameters indicating good mixing of the chains and the observed PSRF was 1.00. Convergence was also visualised in Tracer v1.7.

**Fig. 6:**
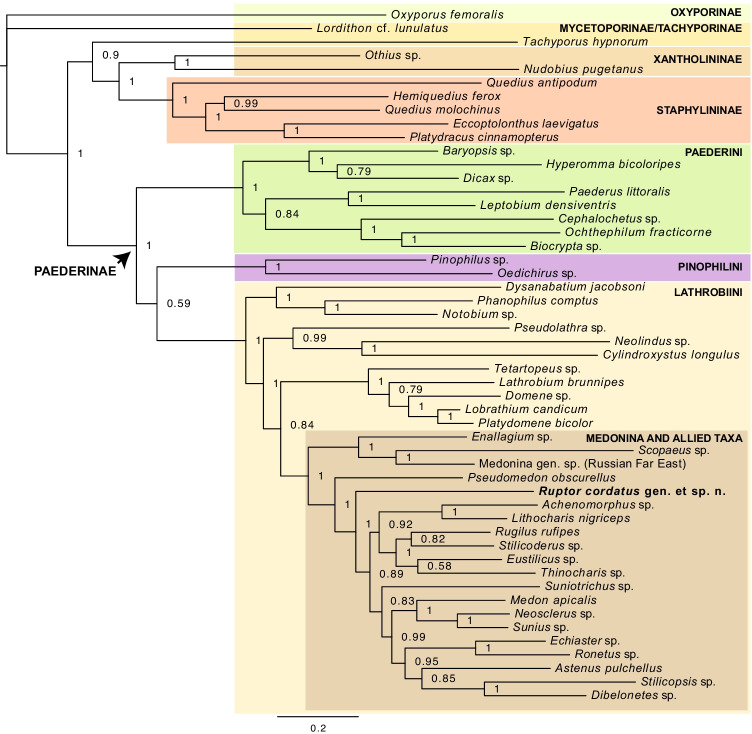
Bayesian Inference (BI) 50% majority rule consensus phylogenetic tree. Nodes with (BI) posterior probability (PP) > 0.95 were considered well supported, nodes with PP = 0.90–0.94 moderately supported, those with PP = 0.80–0.89 weakly supported and nodes with PP < 0.79 were considered to be unsupported

The tree topology presented in Fig. 6 is the 50% majority rule consensus tree. The subfamily Paederinae was recovered as monophyletic with strong support (posterior probability PP = 1) as well as Paederini, Pinophilini and Lathrobiini, its three currently recognised tribes (PP = 1 in all cases). Within Lathrobiini, the first clade (*Dysanabatium* Bernhauer, 1915 + (*Notobium* Solsky, 1864 + *Phanophilus* Sharp, 1886)), all three genera currently classified in Lathrobiina, was well-supported (PP = 1). The next clade (*Pseudolathra* Casey, 1905 + (*Neolindus* Scheerpeltz, 1933 + *Cylindroxystus* Bierig, 1943)) was the *Pseudolathra*-Cylindroxystina lineage recently discovered in Żyła et al. (2021), which was well-supported here too (PP = 0.99). The next resolved clade (*Tetartopeus* Czwalina, 1888 + (*Lathrobium* Gravenhorst, 1802 + (*Domene* Fauvel, 1873 + (*Lobrathium* Mulsant & Rey, 1878 + *Platydomene* Ganglbauer, 1895)))) was the so-called ‘true’ Lathrobiina (PP = 1), recovered as sister to the ‘Medonina and allied taxa’ clade (sensu Żyła et al. 2019) with weak support (PP = 0.84). Within the ‘Medonina and allied taxa’ clade, the clade (*Enallagium* Bernhauer, 1915 [Lathrobiina] + (*Scopaeus* Erichson, 1839 [Scopaeina] + unidentified specimen of a Medonina from the Far East Russia)) branched off first with strong support (PP = 1). The subtribe Medonina was recovered as non-monophyletic, where its bigger fraction formed a group largely paraphyletic with respect to other subtribes in this clade, i.e. Scopaeina, Stilicina, Astenina, Stilicopsina and Echiasterina. *Rugilus* Leach, 1819 + *Stilicoderus* Sharp, 1889 (both Stilicina) and *Eustilicus* Sharp, 1886 (Stilicina) + *Thinocharis* Kraatz, 1859 (Medonina) were altogether recovered as a strongly supported clade (PP = 1). *Dibelonetes* Sahlberg, 1847 and *Stilicopsis* Sachse, 1852 (both Stilicopsina), as well as *Echiaster* Erichson, 1839 and *Ronetus* Blackwelder, 1943 (both Echiasterina) were resolved as monophyletic clades (PP = 1 in both cases), with Stilicopsina sister to *Astenus* Dejean, 1833 (Astenina) (PP = 0.85). Our new genus was recovered as deeply nested within the ‘Medonina and allied taxa’ clade. There, it was resolved as sister to a heterogeneous clade formed by the majority of sampled members of Medonina (all, except for Medonina from Far East Russia and *Pseudomedon* Mulsant & Rey, 1878) and all sampled Scopaeina, Stilicina, Astenina, Stilicopsina and Echiasterina.

## Discussion

Our molecular phylogeny confirmed the placement of a new taxon as a new genus in the tribe Lathrobiini, which could be suspected from its morphology. It was resolved within the informal ‘Medonina and allied taxa’ clade, which can be additionally confirmed by the following morphological characters that *Ruptor* gen. nov. shares with all other members of this clade: small and acicular apical maxillary palpomere and the presence of apical ctenidium on the one side of metatibia only. The position of the new genus within this clade is rather isolated, which, given the currently available subtribal classification, would require erecting a new subtribe for placing *Ruptor* gen. nov. in the classification. However, as shown by the phylogeny here and earlier (Żyła et al. 2019, 2021), the current supra-generic classification of Paederinae needs a revision in order to align it with the phylogenetic data. To avoid the creation of a new family-group name while pending for such a revision, here the new genus is placed in Paederinae as Lathrobiini *incertae sedis*. Based on the distribution of its host and difficulty to collect even a few specimens of *Ruptor* that we have experienced, we presume it may have a broader distribution, potentially with more species to be discovered.

The derived morphology of *Ruptor* gen. nov., with its general compact appearance, highly reduced eyes, short, stout legs and short antennae suggest a degree of co-evolution with *L. labralis*. The fact that *Ruptor* gen. nov. was hitherto not found in any other situation typical of rove beetles (e.g. in leaf litter or in flight intercept traps) further supports its close association and some level of co-evolution with the termite host, in whose nests apparently it spends most of the time.

The overall tree topology in Fig. 6 was consistent with the previous analysis of Żyła et al. (2021), which served as a basis for the molecular-based phylogenetic analysis that we performed here. The most important differences were the position of the tribe Pinophilini which was resolved as sister to Lathrobiini + Paederini in Żyła et al. (2021), but as sister to Lathrobiini in our analysis, and the position of the *Pseudolathra* + Cylindroxystina clade resolved here as sister to Lathrobiina + ‘Medonina and allied taxa’, while it was recovered as sister to ‘Medonina and allied taxa’ clade in Żyła et al. (2021). These differences are most likely a result of the reduced taxon sampling here and the lack of the morphological partition that was included in the previous study. While there were also some local differences in the tree topology within the ‘Medonina and allied taxa’ clade itself, consistently with other studies (Żyła et al. 2019, 2021; Bogri et al. 2020), we recovered Medonina, the largest subtribe within this lineage, as non-monophyletic.

## Supplementary Information

Below is the link to the electronic supplementary material.

Supplementary file1 (JPG 2132 KB)

Supplementary file2 (FAS 233 KB)

Supplementary file3 (NEX 230 KB)

Supplementary file4 (DOCX 25 KB)
