# Supplementary figures and images for: A New Termitophilous Genus of Paederinae Rove Beetles (Coleoptera, Staphylinidae) from the Neotropics and Its Phylogenetic Position
Source: Neotrop Entomol. 2022 Feb 17;51(2):282–91. doi: 10.1007/s13744-022-00946-x (PMC8967768; doi:10.1007/s13744-022-00946-x)

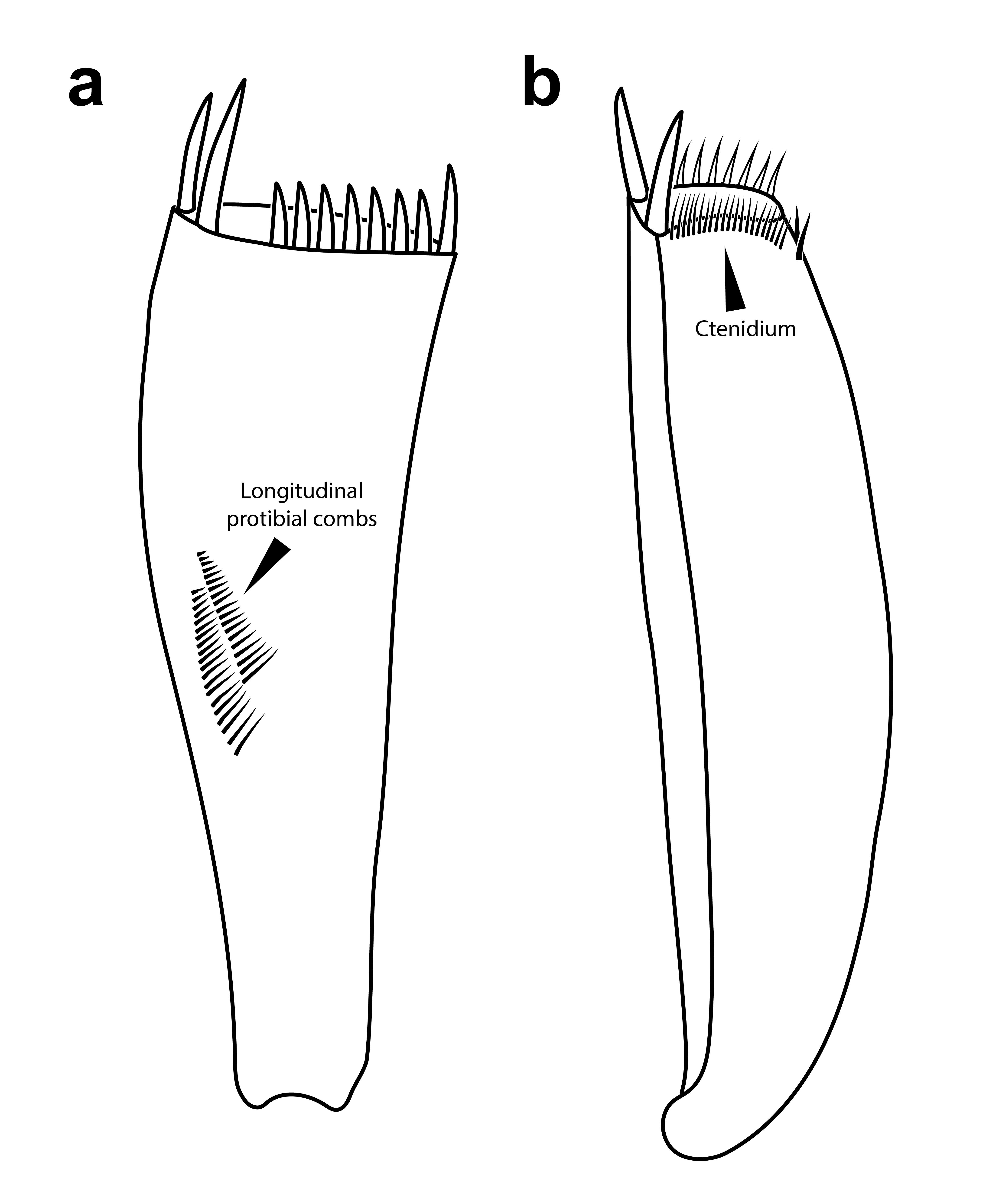

Supplement: Supplementary file 1 — Supplementary file1 (JPG 2132 KB) [file 13744_2022_946_MOESM1_ESM.jpg]
