# Supplementary material for: A New Termitophilous Genus of Paederinae Rove Beetles (Coleoptera, Staphylinidae) from the Neotropics and Its Phylogenetic Position
Source: Neotrop Entomol. 2022 Feb 17;51(2):282–91. doi: 10.1007/s13744-022-00946-x (PMC8967768; doi:10.1007/s13744-022-00946-x)
Supplement: Supplementary file 4 — Supplementary file4 (DOCX 25 KB) [file 13744_2022_946_MOESM4_ESM.docx]

A new termitophilous genus of Paederinae rove beetles (Coleoptera, Staphylinidae) from the Neotropics

Neotropical Entomology

Dagmara Żyła^1,2*^, Amalia Bogri^3^, Aslak Kappel Hansen^3^, Josh Jenkins Shaw^3,4^, Janina Kypke^3^, Alexey Solodovnikov^3^

^1^Museum and Institute of Zoology, Polish Academy of Sciences, Warsaw, Poland

^2^Leibniz Institute for the Analysis of Biodiversity Change, Zoological Museum, Hamburg, Germany

^3^Natural History Museum of Denmark, University of Copenhagen, Copenhagen, Denmark

^4^Key Laboratory of Zoological Systematics and Evolution, Institute of Zoology, Chinese Academy of Sciences, Beijing, China

^*^Corresponding authors: [zyladagmara@gmail.com](mailto:zyladagmara@gmail.com), asolodovnikov@snm.ku.dk

Supplementary Table 1. Material and data used for the molecular phylogenetic analysis. The novel sequences are marked in red. Missing data are indicated with a dash ‘-’.

**MUSM** – Museo de Historia Natural de la Universidad Nacional Mayor de San Marcos

**NHMD** – Natural History Museum of Denmark, Copenhagen (formerly ‘ZMUC’)

**UTCI** – University of Tennessee at Chattanooga

|  | | **Genbank accession numbers** | | | | | |
| --- | --- | --- | --- | --- | --- | --- | --- |
| **Taxon and its systematic placement before present study** | **Depository, Specimen #** | **28S** | **ArgK** | **CADA+CADC** | **COI** | **TP** | **Wg** |
| **Mycetoporinae** | | | | | | | |
| *Lordithon* cf. *lunulatus* (Linnaeus, 1767) | NHMD, NHMD_DZ-10.6 | MN165645 | MN251775 | MN256442 (A) | MN264591 | MN284886 | MN328041 |
| **Tachyporinae** |  |  |  |  |  |  |  |
| *Tachyporus hypnorum* (Fabricius, 1775) | NHMD, NHMD_DZ-7.0 | MN073526 | MN194113 | MN194127 (A) | MN194140 | MN194152 | MN194165 |
| **Oxyporinae** | | | | | | | |
| *Oxyporus femoralis* Gravenhorst, 1802 | UTCI, SC-0192 | KT149213 | KT021925 | KT000243 | KT021973 | KT022020 | KT022070 |
| **Staphylininae** | | | | | | | |
| **Staphylinini** | | | | | | | |
| *Hemiquedius ferox* (LeConte, 1878) | NHMD, 00046184 | KR559838 | KT021915 | KT000233 | KT021963 | KT022016 | KT022039 |
| *Eccoptolonthus laevigatus (*Fauvel, 1895) | NHMD, HSsp01 | GU377330 | KT021914 | KT000232 | GU377374 | GU377425 | GU377476 |
| *Platydracus cinnamopterus* (Gravenhorst, 1802) | UTCI, SC-0081 | GU377342 | KT021920 | KT000239 | GU377389 | GU377440 | GU377491 |
| *Quedius molochinus* (Gravenhorst, 1806) | NHMD, QUmol01 | GU377348 | KF178783 | KF178814 | GU377396 | GU377447 | GU377498 |
| **Xantholininae** |  |  |  |  |  |  |  |
| **Othiini** | | | | | | | |
| *Othius* Stephens, 1829 (indet. sp., Japan) | UTCI, SC-0191 | KR559858 | KT021884 | KT000201 | KT021975 | KT021989 | KT022058 |
| **Xantholinini** |  |  |  |  |  |  |  |
| *Nudobius pugetanus* Casey, 1906 | UTCI, SC-0105 | GU377335 | KT021880 | KT000198 | GU377381 | GU377432 | GU377483 |
| **Paederinae** | | | | | | | |
| **Pinophilini** | | | | | | | |
| Proccirina | | | | | | | |
| *Oedichirus* Erichson, 1839 (indet. sp., Australia) | NHMD, zmuc00046231 | MN073519 | KR259669 | MT809527(A) /  KR259788 (C) | KR259767 | KR259736 | KR259704 |
| Pinophilina | | | | | | | |
| *Pinophilus* Gravenhorst, 1802 (indet. sp., Australia) | NHMD, zmuc00046227 | MT801125 | KR259681 | MN194125 (A)  /  KF178807 (C) | GU377362 | GU377412 | GU377463 |
| **Paederini** | | | | | | | |
| Cryptobiina | | | | | | | |
| *Biocrypta* Casey, 1905 (indet. sp., Mexico) | NHMD, NHMD_DZ-17.3 | MT801101 | MT801159 | MT809517 (C) | MT792650 | MT809534 | - |
| *Cephalochetus myrmecocephalus* (Lea, 1927) | NHMD, zmuc00046234 | - | KR259679 | KR259792 (C) | KR259761 | KR259730 | KR259712 |
| *Cephalochetus* Kraatz, 1859 (indet. sp., Australia) | NHMD_AB-52 & DDM1178 | MT801102 | - | KJ845311 (A) | - | - | - |
| *Ochthephilum fracticorne* (Paykull, 1800) | NHMD, zmuc00046225 & NHMD_AB-55 | MT801119 | KR259663 | MT809526 (A) /  KR259793 (C) | KR259764 | KR259731 | KR259714 |
| Dicaxina | | | | | | | |
| *Baryopsis* Fairmaire & Germain, 1862 (indet. sp., Bolivia) | NHMD, zmuc00046224 & NHMD_AB-49 | MT801100 | MT801158 | KR259783 (C) | MT792649 | KR259729 | KR259693 |
| *Dicax* Fauvel, 1878 (indet. sp., Australia) | NHMD, zmuc00046232 | - | KR259668 | KR259784 (C) | KR259762 | KR259732 | KR259694 |
| *Hyperomma bicoloripes* Schomann, 2014 | NHMD, zmuc00046255 & DDM1149 | KJ844900 | KR259684 | KR259786 (C) | KR259773 | KR259734 | KR259700 |
| *Hyperomma* Fauvel, 1878 (indet. sp., Tasmania) | NHMD,  NHMD_JK-IV46.H | - | - | MT809522 (A) | - | - | - |
| Dolicaonina | | | | | | | |
| *Leptobium densiventris* (Fauvel, 1875) | NHMD, zmuc00046226 | - | KR259683 | KR259785 (C) | KR259763 | KR259726 | KR259708 |
| Paederina | | | | | | | |
| *Paederus littoralis* Gravenhorst, 1802 | NHMD,  NHMD_DZ-20.5 & DDM1166 | MT801122 | - | KJ845307 (A) / MT809528 (C) | MT792667 | MT809547 | MT809573 |
| **Lathrobiini** | | | | | | | |
| Astenina | | | | | | | |
| *Astenus pulchellus* (Heer, 1839) | NHMD, zmuc00046222 | MT801099 | KR259662 | KR259774 (C) | KR259751 | KR259718 | KR259689 |
| Cylindroxystina | | | | | | | |
| *Cylindroxystus* *longulus* Bierig, 1943 (CostaRica) | NHMD, NHMD_DZ-5.3 | MT801105 | MT801161 | MT809502 | MT792653 | - | MT809557 |
| *Neolindus* Scheerpeltz, 1933 (indet. sp., Peru) | MUSM,  NHMD_DZ-NEO | MT801117 | - | MT809524 (C) | MT792664 | MT809543 | MT809569 |
| Echiasterina | | | | | | | |
| *Echiaster* Erichson, 1839 (indet. sp., Costa Rica) | NHMD, NHMD_DZ-8.0 | MN073512 | MN158714 | MN194117 (A) | MN194132 | MN194145 | MN194157 |
| *Ronetus* Blackwelder, 1943 (indet. sp., Mexico) | NHMD,  NHMD_DZ-23.3 | MT801128 | MT801175 | MT809529 (A) | MT792671 | MT809551 | MT809577 |
| Lathrobiina | | | | | | | |
| *Domene* Fauvel, 1873 (indet. sp., Czech Republic) | NHMD, NHMD_DZ-4.1 | MN073511 | MN158713 | MN194116 (A) / MT809520 (C) | MN194131 | MN194144 | MN194156 |
| *Dysanabatium jacobsoni* Bernhauer, 1915 | NHMD, NHMD_AB-1 | MT801108 | MT801163 | MT809503 | MT792655 | MT809537 | MT809560 |
| *Enallagium* Bernhauer, 19l5 (indet. sp., Myanmar) | NHMD, NHMD_AB-23 | MT801110 | MT801165 | MT809504 | MT792657 | MT809539 | MT809562 |
| *Lathrobium brunnipes* (Fabricius, 1792) | NHMD, zmuc00046221 | MN073515 | KR259675 | MT809523 (A) /  KR259779 (C) | KR259757 | KR259722 | KR259709 |
| *Lobrathium candicum* Bordoni, 2009 | NHMD, NHMD_AB-38 | MT801115 | MT801169 | MT809507 | MT792662 | - | MT809567 |
| *Notobium* Solsky, 1864 (indet. sp., Australia) | NHMD, zmuc00046223 | MN073524 | KR259660 | MT809525 (A) /  KR259781 (C) | KR259756 | KR259724 | KR259702 |
| *Phanophilus comptus* (Broun, 1880) | NHMD, zmuc00046230 | MT801124 | KR259661 | KR259780 (C) | KR259755 | KR259723 | KR259705 |
| *Platydomene bicolor* (Erichson, 1840) | NHMD, NHMD_AB-48 | MT801126 | MT801173 | MT809511 | MT792669 | MT809549 | MT809575 |
| *Tetartopeus* Czwalina, 1888 (indet. sp., Czech Republic) | NHMD,  NHMD_DZ-6.1 | MN073527 | MN194114 | MN194128 (A) / MT809533 (C) | MN194141 | MN194153 | MN194166 |
| Medonina | | | | | | | |
| *Achenomorphus* Motschulsky, 1858 (indet. sp., Costa Rica) | NHMD, NHMD_DZ-9.0 | MN073509 | MT801157 | MN194115 (A) / MT809516 (C) | MN194130 | MN194143 | MN194155 |
| *Lithocharis nigriceps* Kraatz, 1859 | NHMD, NHMD_DZ-4.2 | MT801114 | MT801168 | MT809506 | MT792661 | MT809541 | MT809566 |
| *Medon apicalis* (Kraatz, 1857) | NHMD, zmuc00046233 | MN073517 | KR259674 | KR259775 (C) | KR259753 | KR259719 | KR259690 |
| *Neosclerus* Cameron, 1924 (indet. sp., Myanmar) | NHMD, NHMD_AB-34 | MT801118 | MT801171 | MT809509 | MT792665 | MT809544 | MT809570 |
| *Pseudomedon obscurellus* (Erichson, 1840) | NHMD,  NHMD_DZ-14.5 | MT801127 | MT801174 | MT809512 | MT792670 | MT809550 | MT809576 |
| *Sunius melanocephalus* (Fabricius, 1792) | NHMD, NHMD_DZ-5.2 | MN073525 | MN194112 | MN194126 (A) / MT809532 (C) | MN194139 | MN194151 | MN194164 |
| *Suniotrichus* Sharp, 1886 (indet. sp., Mexico) | NHMD,  NHMD_DZ-21.3 | MT801130 | MT801177 | MT809514 | MT792673 | MT809552 | MT809579 |
| *Thinocharis* Kraatz, 1859 (indet. sp., Laos) | NHMD, NHMD_AB-4 | MT801132 | MT801179 | MT809515 | MT792675 | MT809554 | MT809581 |
| Scopaeina | | | | | | | |
| *Scopaeus* Erichson, 1839 (indet. sp., Australia) | NHMD, zmuc00046219 & DDM0325 | MN073523 | KR259680 | KJ845232 (A) /  KR259778 (C) | KR259759 | KR259725 | KR259713 |
| Stilicina | | | | | | | |
| *Eustilicus* Sharp, 1886 (indet. sp., Costa Rica) | NHMD, NHMD_DZ-8.4 | MT801111 | MT801166 | MT809505 | MT792658 | MT809540 | MT809563 |
| *Rugilus rufipes* Germar, 1836 | NHMD, zmuc00046228 & NHMD_JK-VII86.Rr | MN073522 | KR259673 | MT809530 (A) / KR259776 (C) | KR259754 | KR259720 | KR259691 |
| *Stilicoderus* Sharp, 1889 (indet. sp., Australia) | NHMD,  NHMD_DZ-24.6 | MT801129 | MT801176 | MT809513 | MT792672 | - | MT809578 |
| Stilicopsina | | | | | | | |
| *Dibelonetes* Sahlberg, 1847 (indet. sp., Australia) | NHMD, zmuc00046229 | MN073510 | KR259672 | MT809518 (A) /  KR259777 (C) | KR259752 | KR259721 | KR259692 |
| *Stilicopsis* Sachse, 1852 (indet. sp., Mexico) | NHMD,  NHMD_DZ-20.3 | MN165651 | MN251780 | MT809531 (A) / MN256445 (C) | MN264597 | MN284890 | MN328046 |
| Lathrobiini insertae sedis | | | | | | | |
| Medonina gen. and sp. indet. (Russia) | NHMD, NHMD_DZ-2.2 | MT801116 | MT801170 | MT809508 | MT792663 | MT809542 | MT809568 |
| *Pseudolathra* Casey, 1905 (indet. sp., Laos) | NHMD, PAE01 | GU377340 | KF178776 | KF178808 (A) | GU377386 | GU377437 | GU377488 |
| *Ruptor cordatus* gen.. nov. et sp. nov. | MUSM, | OK350469 | OK352263 | OK352262 (A) OK352261 (C) | - | OK352260 | OK352259 |
